# The impact of laronidase treatment in otolaryngological manifestations of patients with mucopolysaccharidosis^☆^

**DOI:** 10.1016/j.bjorl.2015.09.006

**Published:** 2015-12-17

**Authors:** Ana Paula Fiuza Funicello Dualibi, Ana Maria Martins, Gustavo Antônio Moreira, Marisa Frasson de Azevedo, Reginaldo Raimundo Fujita, Shirley Shizue Nagata Pignatari

**Affiliations:** a Universidade Federal de São Paulo (EPM–UNIFESP), Escola Paulista de Medicina, Departamento de Otorrinolaringologia e Cirurgia de Cabeça e Pescoço, São Paulo, SP, Brazil; b Universidade Federal de São Paulo (EPM–UNIFESP), Escola Paulista de Medicina, Departamento de Pediatria, São Paulo, SP, Brazil

**Keywords:** Mucopolysaccharidosis I, Laronidase, Enzymatic replacement therapy, URT infections, Sleep apnea, Hearing loss, Mucopolissacaridose I, Laronidase, Terapia de reposição enzimática, Infecções do TRS, Apneia do sono, Perda auditiva

## Abstract

**Introduction:**

Mucopolysaccharidosis (MPS) is a lysosomal storage disease caused by deficiency of α-l-iduronidase. The otolaryngological findings include hearing loss, otorrhea, recurrent otitis, hypertrophy of tonsils and adenoid, recurrent rhinosinusitis, speech disorders, snoring, oral breathing and nasal obstruction.

**Objective:**

To evaluate the impact of enzymatic replacement therapy with laronidase (Aldurazyme^®^) in patients with mucopolysaccharidosis (MPS I), regarding sleep and hearing disorders, and clinical manifestations in the upper respiratory tract (URT).

**Methods:**

Nine patients with MPS I (8 Hurler-Scheie, and 1 Scheie phenotypes) of both sexes, ages ranging between 3 and 20 years, were included in this study. Patients were evaluated between seven and 11 months before the treatment and between 16 and 22 months after the onset of the enzymatic replacement. They were all submitted to a clinical and otolaryngological evaluation, including nasofibroscopical, polysomnographic and audiologic exams.

**Results:**

The results’ data showed decreasing of the frequency of ear, nose and throat infections, with improvement of the rhinorrhea and respiratory quality. No remarkable changes were observed regarding macroglossia and tonsil and adenoid hypertrophy. Audiometric and polysomnographic evaluations did not show statistical significance.

**Conclusion:**

Enzymatic replacement therapy in patients with mucopolysaccharidosis I provides control of recurrent URT infections, rhinorrhea and respiratory quality, however it is does not seem to improve audiologic and polisomnographic parameters, with no effect on adenoid and tonsils hypertrophy and macroglossia.

## Resumo

**Introdução:**

Mucopolissacaridose (MPS) é uma doença de depósito lisossômico causada pela deficiência de α-l-iduronidase. Os achados otorrinolaringológicos incluem perda auditiva, otorreia, otites de repetição, hipertrofia adenotonsilar, rinossinusite recorrente, distúrbios da fala, roncos, respiração bucal e obstrução nasal.

**Objetivo:**

Avaliar o impacto da terapia de reposição enzimática com laronidase (Aldurazyme^®^) em pacientes com mucopolissacaridose I (MPS I) em relação ao sono, distúrbios auditivos e manifestações clínicas do trato respiratório superior (TRS).

**Método:**

Nove pacientes com MPS I (oito com fenótipo Hurler-Scheie e um com fenótipo Scheie), de ambos os sexos, com idades variando entre 3 e 20 anos, foram incluídos neste estudo. Os pacientes foram avaliados entre 7 e 11 meses antes do tratamento e entre 16 e 22 meses após o início da reposição enzimática. Todos foram submetidos a uma avaliação clínica e otorrinolaringológica, incluindo nasofibroscopia, polissonografia e exames radiológicos.

**Resultados:**

Os dados dos resultados mostraram diminuição da frequência de infecções de orelha, nariz e garganta, com melhora da rinorreia e da qualidade respiratória. Mudanças significativas não foram observadas em relação à macroglossia e à hipertrofia adenotonsilar. Avaliações audiométricas e polissonográficas não apresentaram significância estatística.

**Conclusão:**

A terapia de reposição enzimática em pacientes com mucopolissacaridose I fornece controle de infecções recorrentes do TRS, rinorreia e qualidade respiratória, porém, não parece melhorar os parâmetros audiológicos e polissonográficos, ou exercer efeito sobre a hipertrofia adenotonsilar e macroglossia.

## Introduction

Mucopolysaccharidosis (MPS) is a lysosomal storage disease caused by deficiency of an enzyme involved in the degradation of glycosaminoglycans (GAGs). They are classified according to the involved enzyme and GAG in seven types: I (Hurler, Hurler Scheie and Scheie), II (Hunter), III (Sanfillipo), IV (Morquio), VI (Maroteaux Lamy), VII (Sly) e IX (Natowicz).^1^, ^2^, ^3^

MPS I is caused by deficiency of α-l-iduronidase, which leads to intralysosomal deposits of dermatan and heparan sulfate. It is an autosomic recessive genetic disease, with estimated incidence varying from 1:100,000 for severe cases to 1:800,000 for cases with mild manifestations.^4^

Clinical manifestations of MPS I are extremely heterogeneous, with symptoms that evolve in many ways, from very mild manifestations of late development, without cognitive disorders, and high life time expectation (Scheie), to very severe cases of early onset, rapidly progressive, with neural degeneration and limited capabilities in life, usually manifested by the first decade (Hurler), and passing through an intermediary level of severity (Hurler–Scheie).^1^, ^2^, ^5^

The disease may involve nervous, skeleton, digestive, cardiac, superior and inferior respiratory systems presenting different levels of severity in an independent manner. Regarding the otolaryngological findings, the most frequent symptoms include hearing loss, otorrhea, recurrent otitis, hypertrophy of tonsils and adenoid, recurrent rhinosinusitis, speech disorders, snoring, oral breathing and nasal obstruction.^1^, ^2^, ^6^

Obstructive sleep apnea and hypopnea syndrome (OSAHS) is frequently diagnosed in MPS I patients. Obstructive and restrictive factors such as reduction of the thoracic volume (musculoskeletal alterations), restriction of the diaphragmatic movement due to hepatosplenomegaly, presence of atelectasis secondary to the reduction of the lung volume, deposit of GAGs into the pulmonary interstitial tissue, tracheal stenosis, vocal cord thickening, adenoid and tonsil hypertrophy, macroglossia, short neck, thickened high positioned epiglottis, presence of abundant thick nasal mucous, and limited mouth opening are mainly responsible for the respiratory disorders.^6^, ^7^, ^8^, ^9^, ^10^, ^11^ Hearing loss is also common in patients with MPS I. Although the nature of hearing loss may be conductive, sensorineural or mixed, conductive hearing loss is more frequent and can be explained by several factors, such as the thickening of the middle ear mucus and eardrum produced by deposits of GAGs, Eustachian tube obstruction, ossicular chain malformation, hypopneumatization of the temporal bone and presence of thick copious mucus. Mechanisms to explain the sensorineural hearing loss are unclear. It is believed that a progressive hyperplasia of the arachnoid membrane can compress the cochlear nerve, and that the storage of GAGs inside neurovascular structures of the inner ear, along with alterations of the hairy cells consequent to metabolic disorders usually present in patients with MPS, may be contributory factors.^2^, ^9^, ^12^, ^13^, ^14^, ^15^, ^16^, ^17^

Due to the multisystem involvement, treatment is usually multidisciplinary. Until the 1970s, treatment consisted in palliative methods to improve the quality of life. Currently, the two principal therapeutic tools of MPS I are based on enzymatic replacement (ERT) and transplantation of hematopoietic cells (HCT). The enzymes produced by stem cells from bone marrow or umbilical cord restore to the patient the ability of degradation. Since 1981, more than 200 children have been submitted to this type of treatment. The HCT produces resolution or improvement of OSAHS and conductive hearing loss. The major problem related to this treatment is the high rate of morbidity and mortality (40%), and the difficulty to find compatible bone marrow. Because of the high risk related to this therapy, it is restricted to children with severe disease, preferable before the 18th month of life, when the CNS alterations usually begin to occur.^18^, ^19^, ^20^, ^21^

Laronidase (Aldurazyme^®^) is the enzyme used in MPS I, and it is produced by recombinant DNA technique. Clinical trials with ERT have shown improving of the respiratory status. It was approved by commercial use in 2003, and since then more than 330 patients have been treated.^22^, ^23^, ^24^, ^25^, ^26^ Although improvement of the respiratory conditions is frequently mentioned in the majority of the reports, few studies have addressed the hearing problems and other otolaryngological findings that remain unclear.^27^, ^28^

The individuals included in this study are part of the first clinical trial of treatment of MPS I with laronidase in Brazil.

The objective was to evaluate the impact of ERT with laronidase in respiratory and audiological manifestations of patients with MPS I and clinical manifestations in the upper respiratory tract (URT).

## Method

Nine patients with MPS I (no-Hurler phenotype) were evaluated after approval of the Committee of Ethics in Research (protocol 0337/05).

Each patient received 52 infusions of laronidase, 0.54 mg/kg/dose.

They were all submitted to at least two otolaryngological evaluations, including nasofibroscopic, polysomnographic and audiological examination, between seven and 11 months before and between 16 and 22 months after the beginning of the treatment.

Tonsils 3 and 4 according to Brodsky classification, and adenoids occupying more than 70% of the choanae were considered hypertrophic.^29^

The data obtained from polysomnographic examination (PSG) were analyzed according to the parameters defined by the American Thoracic Society (index of apnea hipopnea >1 event/h in children under 14 years of age and >5 events/h in older children).^30^, ^31^

Audiological evaluation was accomplished by conventional audiometric exam alone or with visual reinforcement. Analysis of the data was based on SRT (“speech reception threshold”). It was considered normal when the SRT was < or equal 20 (dB). Imitanciometric curve was interpreted according to Jerger classification (1970).^32^, ^33^

Statistical evaluation was analyzed by the Friedman test (non-parametric). The value of rejection for hypothesis of nullity was fixed for values < or equal 0.05. Significance was marked as (*). Non-significant (NS).

## Results

From the nine patients initially evaluated, two did not return for the second visit after the beginning of treatment and were not included in the statistical analysis. Four patients were female and five male, and ages ranged between 3 and 20 years (median of 8 years).

One patient did not have a pre-treatment polysomnographic exam and was not included in the final polysomnographic evaluation.

Three patients had undergone ENT surgery before the start of this study: in addition to myringotomy with ventilating tube placement, one patient underwent adenoidectomy at the age of 7, one patient was referred to adenotonsillectomy at the age of 4 and another patient held elective tracheotomy and adenoidectomy at 4 years of age.

**Figure 1:**
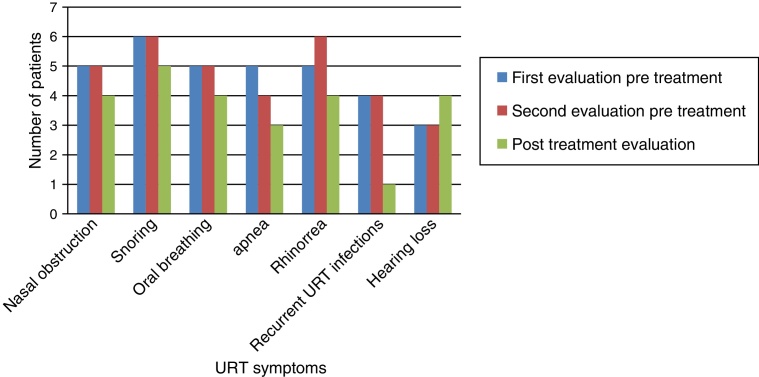
Otolaryngological symptoms obtained from directed anamnesis.

Fig. 1 shows that nasal obstruction, snoring, oral breathing, apnea, rhinorrhea, and recurrent URT infections improved with ERT in practically all patients.

**Figure 2:**
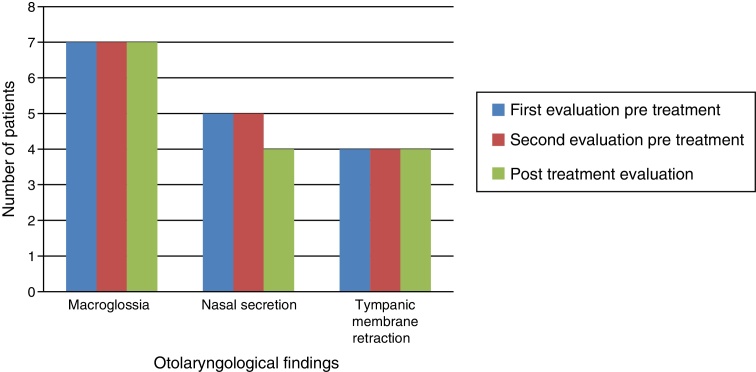
Otolaryngological findings.

Otolaryngological examination showed that macroglossia and appearance of the tympanic membrane (retraction) did not change after ERT. In the three patients without tympanic membrane retraction, two had patent ventilating tubes in place. All patients experienced reduction of nasal mucus after ERT (Fig. 2).

**Figure 3:**
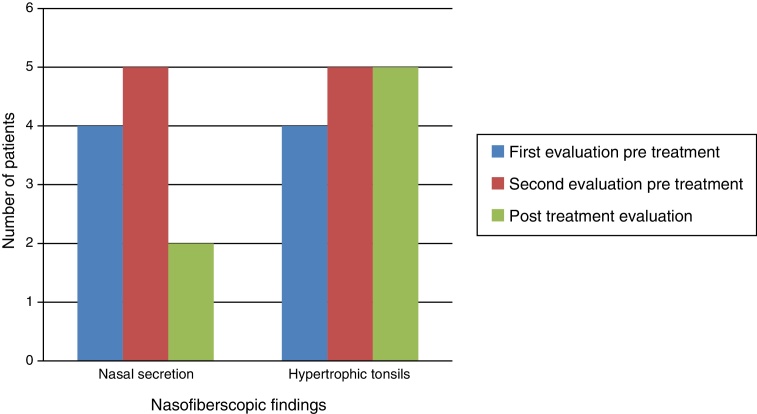
Major findings at the naso-fiberoscopy examination.

Naso-fiberscopic examination also showed reduction of the nasal secretion, however, reduction of tonsil size was not observed (Fig. 3).

The results obtained by audiometric evaluation are shown in Table 1. Statistical analysis did not demonstrate significance of the differences between pre- and post-ERT.

**Table 1:** Results of STR (dB).

Patients	1st evaluation pre treatment		2nd evaluation pre treatment		3rd evaluation post treatment	
	RE	LE	RE	LE	RE	LE
1	30	35	15	20	20	15
2	20	20	25	25	20	30
3	50	60	50	60	65	55
4	55	50	60	35	55	35
5	80	60	90	95	75	80
6	45	50	75	70	80	70
7	75	75	60	60	55	70

Friedman's test: *χ*^2^ calc = 0.240; N.S., *p* = 0.887.

Tympanometry was performed in only five patients, since two had bilateral patent ventilating tubes in place at the moment of the evaluation. In the first evaluation four of them presented B-type curve and one patient presented A-type curve, remaining unchanged after ERT.

Results obtained with polysomnographic exams are shown in Table 2. Statistical analysis did not show significance between the parameters pre- and post-therapy.

**Table 2:** Apnea–hypopnea indexes (IAH) and time of sleep with oxygen saturation inferior to 90%.

Patients	IAH			T sat < 90%		
	1st	2nd	3rd	1st	2nd	3rd
1	10.9	12.3	12.3	2.7	12.5	12.5
2	11.2	1.3	39.3	2	0	7
3	0.3	0	0.4	10	20	32
4	48.1	54.5	60.6	100	91	94
5	9.3	14	10.6	3.4	7	2
6	17.6	15.4	14.1	2.3	0.2	0

Friedman's test.

IAH, *χ*^2^ calc = 1.826; N.S., *p* = 0.401.

Time of sleep with oxygen sat <90%, *χ*^2^ calc = 0.087; N.S., *p* = 0.957.

## Discussion

Since the first description of MPS, at the beginning of the 20th century, the knowledge of this progressive disease of large phenotypic diversity, as well as significant limitation of quality and expectancy of life have expanded considerably. However, despite the remarkable improvement of the understanding of its natural history and treatment, many questions remain unsolved. There is a lack of information regarding the evolution of non-treated patients, and the rarity and large phenotypic heterogenicity of MPS I make the development of a study of clinical relevance difficult, particularly due to the diversity of clinical presentations. The absence of specific scores and biomarkers represents an additional problem to monitor the efficacy of any kind of therapeutic protocol.^4^

Most of the studies enrolling patients with MPS I and ERT have addressed more advanced stages of the disease. The present study is prospective, observational longitudinal, and the patients were already presenting established lesions. This may have contributed to the low expressive results.

Otolaryngological complaints (oral breathing, snoring, hearing loss, nasal obstruction, rhinorrhea, recurrent URT infections) and clinical findings (macroglossia, hypertrophy of tonsils and adenoid, nasal secretion and retraction of tympanic membrane) are frequently reported in the medical literature.^8^, ^9^, ^10^, ^11^, ^12^

In our study, we observed a decrease in frequency of practically all otolaryngologic complains after ERT, with marked reduction in rhinorrhea and URT recurrent infections. We believe that the reduction of the GAG deposits in the middle ear and URT, and consequent improvement of the nasal flow and Eustachian tube could have been the reason for such improvement. Interestingly, hypoacusia frequency increased after ERT.

Most of our results are similar to those reported in the literature. In phase I/II clinical trials, authors have reported 90% of the patients presenting with recurrent URT infections, and improvement after ERT.^23^ Sardón et al. described two patients under ERT, with significant reduction of the tracheotomy secretions in one of the patients after 40 weeks of laronidase, although no improvement of the hypoacusia could be observed.^28^ Tokic et al., in 2007, reported two cases of MPS I submitted to ERT, with remarkable improvement of the URT recurrent infections in both patients, and also improvement of the audiological parameters.^29^

Along the time, naso-fiberoptic examination showed continuous increase of hypertrophy of tonsils and adenoids, as much as nasal secretions, in evaluations before ERT. After the beginning of ERT, nasal secretion showed important reduction, but no changes regarding tonsils’ hypertrophy were observed. There are no references in the literature regarding the volume of the tonsils and adenoids in MPS I patients submitted to ERT. However, some authors performed tonsillectomy and adenoidectomy in at least five patients undergoing ERT, and this fact may reflect the absence of improvement of this clinical parameter.^27^

Regarding the hearing loss, there is a consensus among the reports that MPS I patients present with hypoacusia, and although some authors report improvement after HCT, few have evaluated the evolution of dysacusia after ERT. Sardón et al., in 2005, evaluated two patients performing brain evoked response audiometry (BERA), detecting conductive hearing loss in one. The patient did not present improvement after ERT.^23^ Tokic et al., in 2007, also evaluated two patients hearing by using traditional audiometry. A mixed conductive-sensorineural hearing loss was observed in one patient, with no changes after ERT, and conductive hearing loss was observed in the other, which presented improvement of the thresholds from 30 dB to 10 dB in one ear and 90 dB to 60 dB in the other ear after ERT.^29^

One hundred percent of our patients presented hearing loss of varying levels. Two patients had ventilation tubes in place and were not submitted to tympanometry. Type-B curve was observed in 80% of the remaining five patients. There was no statistical significance of the values between just before and after ERT, however, according to the medians, it was possible to verify that the hearing loss was getting worse before ERT, and had a slight increase after treatment. Along with the improvement of the URT infections and rhinorrhea, a better result regarding the hearing thresholds was also expected, but possibly other factors such as ossicular chain malformation, thickening of the middle ear mucosa, and Eustachian tube dysfunction were responsible for the maintenance of the audiological status. On the other hand, sensorineural hearing losses are usually progressive and tend to worsen the audiologic threshold of MPS I patients, even during ERT, since the enzyme does not cross the hematoencephalic barrier.^17^

When first submitted to polysomnographic examination, all patients presented OSAHS, except one patient who had had tracheostomy before the beginning of the study, and no OSAHS was registered in any occasion. Overall, the results did not show statistical difference between the pre- and post-ERT measurements, however, based on the medians, we observed that the polysomnographic parameters were deteriorating before therapy and presented stabilization after the ERT. This phenomenon was also observed for the medians of the time of oxyhemoglobin under 90%.

According to the literature most of the patients improved their respiratory status after ERT. Phases I/II clinical trials have shown that all patients with OSAHS presented reduction of the IAH after 52 weeks of ERT (mean of 2.1–1 event/h); two patients had reduction of sleep time with oxygen saturation under 90%; one patient had improvement in sleep time. After six years of follow-up, five out of six patients were re-evaluated, four presented improvement or stabilization of the respiratory status, and one patient was worse.^26^

Phase III study also showed reduction of the IAH in patients treated with enzyme when compared to a placebo group (reduction of 6 events/h in the treated group, increase of 0.3 event/h in the placebo group). In a study addressing children under the age of 5 years, reduction of 8.5% of the IAH was observed. However, among six patients who presented normal IAH before treatment, four remained normal and two got worse after therapy.^25^ Tokic et al. also reported normalization to the IAH in two patients after 12 weeks of treatment, along with improvement of the respiratory pattern.^29^

Few isolated reports, however, present contrary results regarding the respiratory quality. Sardón et al. had a patient with tracheotomy who did not show improvement of the respiratory quality after ERT.^23^ Thomas et al. described a severely diseased patient treated with ERT for three years with no changes on the progression of the obstructive picture.^34^

Due to the progressive pattern of MPS I, stabilization or reduction of lesions and speed of the disease progression are considered as benefits from the therapy.^2^, ^26^

When a questionnaire is completed by the patients, studies have shown improvement in daily activities performance. Our patients have also described a general improvement, even regarding the hypoacusia, sleep and respiratory quality, which we could not demonstrate through the results of the objective exams (audiometric and polysomnographic evaluations).

Current challenges for ERT, besides the development of an early diagnostic protocol (neonatal screening test), consist of prediction of the severity of the disease, in order to make an adequate choice of treatment, to develop an adequate treatment for the neuropathies, and to find ways for an effective monitoring, with specific instruments to quantify the improvement of life quality as well as efficacy and effectiveness of the employed therapy. Longer follow-ups can also contribute to the knowledge of the side effects of the ERT as much as the evaluation of the evolution of each system in order to plan associated treatment programs.^2^, ^35^, ^36^

Although this was a study project of a small sampling, overall there was a remarkable improvement of the quality of life of patients and a high level of satisfaction by the relatives and health care professionals produced by the ERT.

## Conclusion

According to the results obtained from this study, we observed that MPS I patients submitted to ERT with laronidase (Aldurazyme^®^) present improvement of the URT recurrent infections, rhinorrhea, and general respiratory status, without however presenting expressive and significant improvement of the hearing loss, tympanometric curve pattern, sleep disorders, macroglossia and tonsils and adenoid hypertrophy.

## Conflicts of interest

The authors declare no conflicts of interest.
